# The association between the dietary inflammatory index and cardiorespiratory fitness in United States young adults: a cross-sectional study from the National Health and Nutrition Examination Study, 1999–2004

**DOI:** 10.3389/fnut.2024.1442710

**Published:** 2024-09-26

**Authors:** Bo Wu, Lanlan Qiu, Yun Lin, Qian Lin, Yuxiong Pan

**Affiliations:** ^1^Department of Cardiology, Longyan First Affiliated Hospital of Fujian Medical University, Longyan, China; ^2^Department of Cardiology, Longyan People’s Hospital, Longyan, China

**Keywords:** dietary inflammatory index, cardiorespiratory fitness, NHANES, inflammatory, cross-sectional study

## Abstract

**Background:**

Cardiorespiratory fitness (CRF) is a vital indicator of overall health and cardiovascular efficiency. Systemic inflammation significantly impacts CRF, and reducing systemic inflammation may serve as an effective strategy to improve CRF. Diet plays a crucial role in systemic inflammation, but daily dietary intake typically involves multiple elements rather than a single nutrient. The Dietary Inflammatory Index (DII) provides an overall assessment of dietary inflammation on the basis of the anti-inflammatory and pro-inflammatory effects of the nutrients consumed. However, the relationship between DII and CRF is not yet well understood.

**Aims:**

To examine the association between the DII and CRF.

**Method:**

This study analyzed 3,087 participants from the National Health and Nutrition Examination Survey (NHANES) between 1999 and 2002. The study subjects were divided into three distinct groups by DII tertile: T1 (*n* = 1,027), T2 (*n* = 1,029), and T3 (*n* = 1,031). The associations between DII levels and CRF were examined via logistic regression analysis and restricted cubic splines (RCSs).

**Results:**

Elevated DII scores were significantly linked to low CRF levels. Compared with those in the lowest tertile, participants in the highest DII tertile exhibited a greater prevalence of low CRF (T1: 10.85%, T2: 16.32%, T3: 19.31%). In the model with full adjustments, elevated scores on the DII were consistently linked with a heightened likelihood of low CRF (OR: 1.17, 95% CI: 1.07–1.28; *P* < 0.001). Compared with those in the T1 group, participants with higher DIIs had an increased risk of lower CRF (T2: OR: 1.42, 95% CI: 1.01–2.01, *P* = 0.046; T3: OR: 1.71, 95% CI: 1.22–2.40, *P* = 0.003). Additionally, a significant interaction (*P* = 0.045) between sex and the DII for low CRF was observed within the population.

**Conclusion:**

A higher DII score is linked to an elevated risk of low CRF. Moreover, sex can impact CRF, with women being more prone to low CRF.

## Introduction

Cardiorespiratory fitness (CRF) is an essential marker of health, indicating the proficiency of the heart and lungs in delivering oxygen to the muscles during exercise (1, 2). Low CRF is a well-recognized predictor of premature mortality and is associated with increased risks of cardiovascular disease (CVD), cancer and type 2 diabetes mellitus (3–5). CRF is a modifiable factor, and improvements in CRF can positively impact the incidence and mortality rates of cardiovascular diseases.

Inflammation is believed to be associated with impaired CRF. Reducing inflammation levels may be a potential method for improving CRF (6, 7). Current research suggests that improving dietary patterns is one way to reduce inflammation in community populations (8, 9). This concept is based on the idea that specific dietary components can either trigger or mitigate physiological inflammation (10). Proinflammatory diets, which are typically high in refined sugars, trans fats, and saturated fats, are associated with increased concentrations of inflammatory biomarkers, including interleukin-6 (IL-6) and tumor necrosis factor-alpha (TNF-α). Conversely, diets rich in vegetables, fruits, whole grains, and omega-3 fatty acids exhibit anti-inflammatory effects (11). Therefore, anti-inflammatory diets represent a potential approach for improving CRF. A study from Japan demonstrated that the intake of certain micronutrients is independently and positively associated with CRF in Japanese men (12). In addition, Farley et al. confirmed that omega-3 polyunsaturated fatty acids modify the inverse association between systemic inflammation and CRF (13).

It is important to recognize that the dietary intake of community populations is not limited to single elements. Even the intake of a single food involves multiple elements. Therefore, it is impractical to consider the impact of individual nutrients on CRF alone; a method for comprehensively assessing dietary inflammatory potential is needed to evaluate dietary inflammation as a whole. Some studies have proposed the dietary inflammatory index (DII) to assess dietary inflammation comprehensively (14). Both epidemiological and clinical studies have consistently demonstrated that high DII scores are correlated with elevated risks of CVD, stroke, and cancer (15). However, it remains unclear whether the overall degree of dietary inflammation assessed by the DII can be used to evaluate the risk of impaired CRF.

By elucidating the relationship between DII and CRF, this study aims to provide insights for improving CRF in community populations with the goal of improving cardiovascular health.

## Materials and methods

### Study design and participants

This investigation adopts a cross-sectional approach, making use of data from the National Health and Nutrition Examination Survey (NHANES), which is a suite of studies aimed at determining the health and nutrition levels of American adults and children. Every participant in the NHANES study provided written consent, and the study methods were authorized by the Institutional Review Board at the Centers for Disease Control and Prevention. Detailed methodologies are available on the official website of the NHANES database.^1^

**FIGURE 1:**
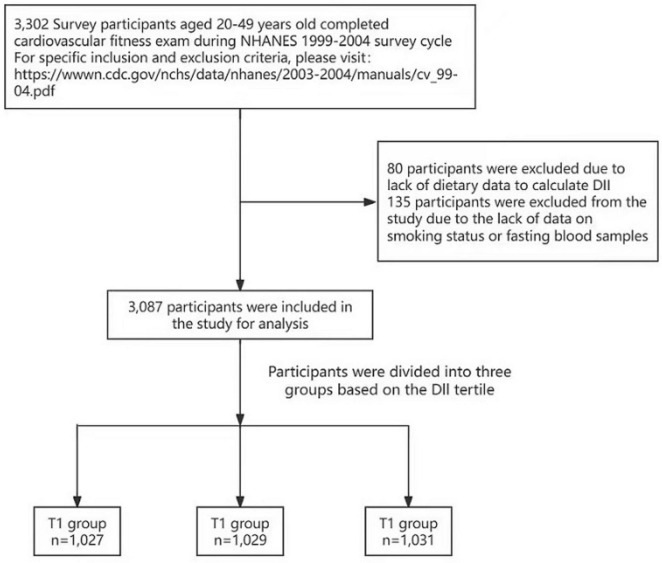
Flowchart of the study design.

The NHANES survey integrates in‘-interviews and physical examinations that address dietary intake and fitness assessments. Our analysis specifically examines the relationship between the DII and CRF within the noninstitutionalized civilian US population. A total of 3,302 survey participants aged 20–49 years completed the CRF exam during the NHANES 1999–2004 survey cycle. Eighty participants were excluded because of a lack of dietary data available to calculate the DII, and 135 participants were excluded from the study because of a lack of data regarding smoking status or fasting blood samples (Figure 1).

We utilized data from the NHANES study conducted between 1999 and 2004 to perform a cross-sectional analysis involving 3,087 participants who underwent CRF examinations. Individuals who participated in the survey were eligible for the CRF tests. During these tests, the participants were generally instructed to perform exercises on a treadmill.

### Fitness estimation

The CRF module of the NHANES, initiated in 1999, is designed to collect comprehensive data representative of the national population on CRF and its correlation with diverse health outcomes. Given the impracticality of conducting maximal exercise tests on a large population across multiple examination sites, a submaximal treadmill exercise protocol was utilized instead.

The classification of CRF levels was determined via cutoffs that were specific to sex and age and were extrapolated from measurements of maximal oxygen consumption (VO2max) in individuals 49 years. The establishment of these thresholds was based on findings from the Aerobics Center Longitudinal Study (ACLS) (16). Comprehensive guidelines for calculating VO2max and detailed information on the protocol are documented in the NHANES Cardiovascular MEC Manual.^2^ In other NHANES-related studies, the calculation of the CRF has also been described in detail (17).

Submaximal exercise assessments are clinically employed for the diagnosis of CVD. However, estimating peak fitness levels via submaximal exercise tests is not optimal, as individuals with high fitness may not need to exert themselves to their fullest potential. Therefore, our report focuses on “Low Cardiorespiratory Fitness” in the population, categorized into low, moderate, and high fitness categories on the basis of reference percentiles as recommended in the NHANES fitness assessment manual. Low fitness corresponds to the 20th percentile, moderate fitness ranges from the 20th to 59th percentiles, and high fitness is defined at the 60th percentile and above on the basis of published ACLS data (16, 18). “Moderate fitness” and “high fitness” were defined as “non-low cardiorespiratory fitness”.

### Calculation of the DII

In this study, the calculation of the DII is consistent with that used by Shivappa et al. (19). This method has also been used in other studies utilizing the NHANES database (20). The calculation of DII employs six important systemic inflammation markers (IL-1β, IL-6, IL-4, IL-10, TNF-α, and CRP) to reflect inflammation levels. Dietary components were assigned “+1” if they increased the levels of CRP, TNF-α, IL-1β, and IL-6 or reduced the levels of IL-4 and IL-10; if a dietary component decreased the levels of CRP, TNF-α, IL-1β, and IL-6 or increased the levels of IL-4 and IL-10, “−1” was assigned. For each individual food parameter, this score was multiplied by the respective food parameter effect score derived from the literature review. All of the food parameter-specific DII scores were then summed to create the overall DII score for each participant in the study: DII = b1 * n1 + b2 * n2……….b28 * n28, where b refers to the literature-derived inflammatory effect score for each of the evaluable food parameters and n refers to the food parameter-specific percentiles, which were computed from the FFQ (food frequency questionnaire)-derived dietary data.

Importantly, in this study, the DII was derived from dietary consumption data. A total of 28 nutrients, including carbohydrates, proteins, fats, alcohol, dietary fiber, cholesterol, saturated fatty acids, monounsaturated fatty acids, polyunsaturated fatty acids, omega-3 and omega-6, niacin, a range of vitamins (A, B1, B2, B6, B12, C, D, E), and several minerals and compounds (iron, magnesium, zinc, selenium, folic acid, beta-carotene, caffeine), as well as overall caloric intake, were included in the calculations. Dietary information from the NHANES was gathered via a 24-h dietary recall interview conducted at the Mobile Examination Center (MEC). Although the calculation of the Dietary Inflammatory Index (DII) in this study utilized only 28 nutrients, previous research has indicated that using only these 28 nutrients does not compromise the assessment efficacy of the DII (14).

In our study, the DII was evaluated as a continuous measure, with participants being divided into three groups according to their respective DII scores: T1 (DII ≤ 0.68), T2 (0.68 < DII ≤ 2.33), and T3 (DII > 2.33).

### Other measures

The participants self-reported data on age, race/ethnic background, and education, whereas information on diabetes history, drinking status, and smoking habits was obtained from health-related questionnaires. Blood pressure readings were taken three to four times while the participants were seated, and a mercury sphygmomanometer was used for measurement. Following a minimum 8-h overnight fasting period, blood samples were obtained for the evaluation of various biomarkers, including high-density lipoprotein cholesterol (HDL-C), total cholesterol (TC), triglycerides (TG), and hemoglobin electrophoresis (Hb). Detailed procedures for blood biochemical measurements are available on the NHANES website.^3^

Hypertension determination is based on two criteria. First, individuals affirming a physician’s diagnosis of hypertension or the use of antihypertensive medication, as per NHANES inquiries, are classified as hypertensive. Second, for those assessed at the mobile examination center or during home visits, individuals were classified as having hypertension if a systolic blood pressure (SBP) exceeding 140 mmHg or a diastolic blood pressure (DBP) above 90 mmHg were recorded. When multiple blood pressure measurements were available, the average was utilized for hypertension diagnosis. Additionally, the presence of comorbid conditions, including diabetes mellitus (DM) and chronic kidney disease (CKD), was confirmed through positive responses to relevant health queries.

On the basis of the International Physical Activity Questionnaire assessment standards (21), participation in more than 60 min of moderate-to-vigorous physical activity per week was defined as moderate-to-high physical activity, whereas participation in 60 min or less of physical activity per week was defined as low physical activity. Specific data on physical activity also came from participants’ self-reports.

### Statistical analysis

The weights for specific groups were calculated according to the NHANES recommendations. Continuous variables were delineated as mean values with standard errors, and categorical variables were denoted as frequency counts with corresponding percentages. Initial comparisons of baseline traits across diverse groups utilized ANOVA for continuous data and chi-square tests for categorical data. The link between the DII and CRF was examined through logistic regression. Statistical evaluations employed complex sample weighting, adhering to NHANES-prescribed weights. To bolster the validity of our findings, we scrutinized three distinct models. Model 1 was unadjusted. Model 2 was adjusted for race, sex, and age. Model 3 was fully adjusted for potential confounders, including race, sex, age, BMI, DM, hypertension, CKD, smoking status, alcohol consumption status, physical activity, and Hb. Additionally, a regression cubic spline (RCS) analysis was conducted to explore the potential nonlinear relationship between DII and CRF, with adjustment variables consistent with Model 3. Furthermore, we stratified the analysis by age, sex, BMI, alcohol consumption status and physical activity level to examine potential interactions between the DII score and these subgroups.

Data analysis was performed via the survey package within the R programming environment (version 4.2.2, Vienna, Austria). Statistical significance was established at a two-tailed P value threshold of less than 0.05 for all tests conducted.

## Results

The study analyzed data from 3,087 participants in the NHANES 1999–2004, with an average age of approximately 34.03 years, 52.37% of whom were men. The participants were categorized into tertiles on the basis of their DII scores, with T1 comprising 1,027 participants, T2 comprising 1,029 participants, and T3 including 1,031 participants. Significant age differences were observed across tertiles [T1: 34.78 (0.31) years vs. T2: 33.89 (0.29) years vs. T3: 33.39 (0.41) years, *P* = 0.010]. Groups with higher DIIs exhibited a greater proportion of women [T1: 340 (34.27%) vs. T2: 507 (48.31%) vs. T3: 623 (61.20), *P* < 0.001], and a greater proportion of current smokers was also observed [T1: 247 (22.53%) vs. T2: 269 (27.31%) vs. T3: 307 (31.14%), *P* = 0.002]. Additionally, in groups with higher DIIs, the proportions of alcohol consumers [T1: 816 (81.3%) vs. T2: 790 (79.5%) vs. T3: 735 (73.7%), *P* = 0.007] and those with high physical activity [T1: 577 (59.39%) vs. T2: 522 (54.19%) vs. T3: 464 (48.77%), *P* < 0.001] were lower. Significant differences in BMI [T1: 26.57 (0.18) kg/m^2^ vs. T2: 27.37 (0.25) kg/m^2^ vs. T3: 27.36 (0.26) kg/m^2^, *P* = 0.003] were observed among the three groups of participants. No significant differences were observed among the groups with respect to the incidence of hypertension, DM CKD, TG, TC, or HDL. Further details can be found in Table 1.

**TABLE 1:** Baseline characteristics of the study population according to tertiles of the DII (weighted).

Characteristic	Overall *n* = 3,087	T1 group *n* = 1,027	T2 group *n* = 1,029	T3 group *n* = 1,031	*P*-value
Age, years	34.03 (0.20)	34.78 (0.31)	33.89 (0.29)	33.39 (0.41)	0.014
Sex					<0.001
Female	1,470 (47.63)	340 (34.27)	507 (48.31)	623 (61.20)	
Male	1,617 (52.37)	687 (65.73)	522 (51.69)	408 (38.80)	
Race					<0.001
Non-Hispanic Black	593 (10.03)	147 (7.14)	207 (10.76)	239 (12.38)	
Non-Hispanic White	1,465 (70.78)	528 (74.54)	453 (66.64)	484 (70.95)	
Other Race	99 (3.99)	38 (3.95)	34 (4.31)	27 (3.72)	
Mexican American	784 (9.58)	272 (9.74)	265 (9.98)	247 (9.00)	
Other Hispanic	146 (5.62)	42 (4.64)	70 (8.30)	34 (3.94)	
BMI, kg/m2	27.09 (0.14)	26.57 (0.18)	27.37 (0.25)	27.36 (0.26)	0.003
Smoking status					0.002
Never smoker	1,775 (56.39)	593 (58.32)	601 (55.54)	581 (55.18)	
Former smoker	489 (16.72)	187 (19.15)	159 (17.16)	143 (13.68)	
Current smoker	823 (26.90)	247 (22.53)	269 (27.31)	307 (31.14)	
Alcohol consumption, *n* (%)	2,341 (78.2)	816 (81.3)	790 (79.5)	735 (73.7)	0.007
Physical activity					<0.001
Low physical activity	1,524 (45.77)	450 (40.61)	507 (45.81)	567 (51.23)	
High physical activity	1,563 (54.23)	577 (59.39)	522 (54.19)	464 (48.77)	
Hypertension, *n* (%)	411 (13.20)	131 (13.21)	140 (12.64)	140 (13.74)	0.868
DM, *n* (%)	90 (2.25)	21 (1.51)	37 (2.57)	32 (2.72)	0.192
CKD, *n* (%)	159 (4.35)	44 (4.07)	56 (4.42)	59 (4.56)	0.889
TG, mmol/L	1.42 (0.04)	1.49 (0.08)	1.43 (0.06)	1.33 (0.05)	0.245
TC, mmol/L	5.01 (0.02)	4.99 (0.04)	5.04 (0.04)	5.00 (0.04)	0.696
HDL, mmol/L	1.34 (0.01)	1.35 (0.01)	1.35 (0.02)	1.33 (0.02)	0.472
Hb, g/dl	14.62 (0.06)	14.83 (0.08)	14.62 (0.07)	14.39 (0.07)	<0.001
Levels of CRF					<0.001
High CRF	2,572 (84.60)	905 (89.15)	843 (83.61)	824 (80.69)	
Low CRF	515 (15.40)	122 (10.85)	186 (16.32)	207 (19.31)	
DII	1.28 (0.05)	−0.72 (0.03)	1.56 (0.02)	3.13 (0.02)	<0.001

BMI, body mass index; DM, diabetes mellitus; CKD, chronic kidney disease; TG, triglyceride; TC, total cholesterol; HDL-C, high-density lipoprotein cholesterol; Hb, hemoglobin; CRF, cardiorespiratory fitness; DII, dietary inflammatory index.

### Relationship between DII scores and low CRF

**FIGURE 2:**
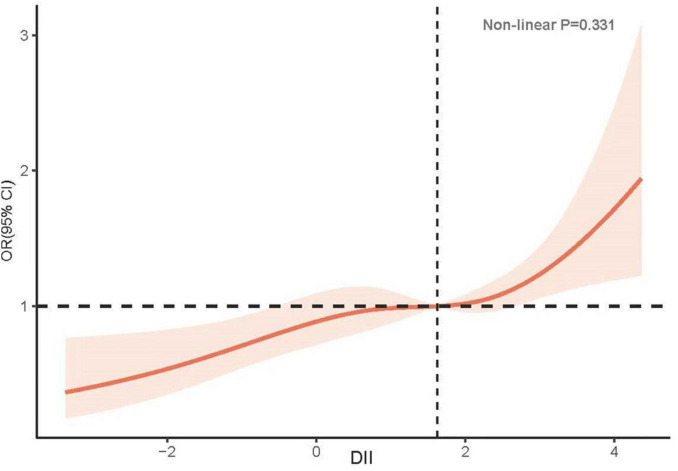
Potential nonlinear relationship between DII and low CRF (weighted).

In unadjusted Model 1, an increase in DII was positively associated with the incidence of low CRF (OR: 1.22, 95% CI: 1.18–1.32; *P* < 0.001). Compared with the T1 group, the T2 group exhibited a greater risk of developing low CRF (T2: OR: 1.60, 95% CI: 1.15–2.22, *P* = 0.006; T3: OR: 1.97, 95% CI: 1.41–2.74, *P* < 0.001). After adjusting for confounding factors, including race, sex, age, BMI, DM, hypertension, CKD, smoking status, alcohol consumption status, physical activity, and Hb, the relationship between DII and low CRF did not significantly change (OR: 1.17, 95% CI: 1.07–1.28, *P* < 0.001). A greater risk of low CRF was observed in the groups with higher DIIs (T2: OR: 1.42, 95% CI: 1.01–2.01, *P* = 0.046; T3: OR: 1.71, 95% CI: 1.22–2.40, *P* = 0.003) (Table 2). The results of the RCS analysis revealed a linear relationship between DII and low CRF (nonlinear *P* = 0.331) (Figure 2).

**TABLE 2:** The association between DII and low CRF.

Variable		Model 1		Model 2		Model 3	
		OR (95% CI)	*P*-value	OR (95% CI)	*P*-value	OR (95% CI)	*P*-value
Continuous variables		1.22 (1.18,1.32)	<0.001	1.20 (1.11,1.30)	< 0.001	1.17 (1.07,1.28)	<0.001
**DII**							
Categorical variable	Event/All population						
T1 group	122/1,027	Ref		Ref		Ref	
T2 group	186/1,029	1.60 (1.15,2.22)	0.006	1.54 (1.12,2.13)	0.010	1.42 (1.01,2.01)	0.046
T3 group	207/1,031	1.97 (1.41,2.74)	<0.001	1.86 (1.34,2.57)	<0.001	1.71 (1.22,2.40)	0.003

Model 1: Not adjusted. Model 2: Adjusted for age, sex, and race. Model 3: Adjusted for race, sex, age, BMI, DM, hypertension, CKD, smoking status, drinking status, physical activity, and Hb.

### Subgroup analysis of DII and the risk of low CRF

**FIGURE 3:**
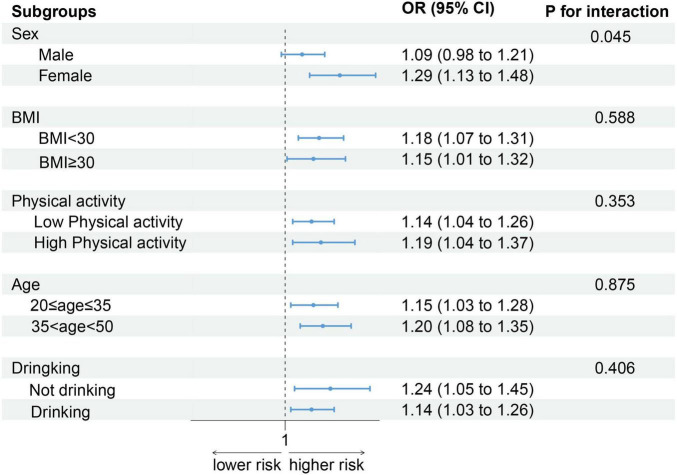
Association between DII and low CRF in selected subgroups (weighted).

As shown in Figure 3, after stratification by sex, BMI, physical activity, age, and alcohol consumption status, the positive association between the DII score and low CRF remained. Notably, an increase in DII had a greater effect on women (OR: 1.29, 95% CI: 1.13–1.48) than on men (OR: 1.09, 95% CI: 0.98–1.21) (*P* for interaction = 0.045).

## Discussion

In this cross-sectional study of 3,087 adults, we identified a significant positive association between higher DII scores and low CRF. This finding suggests that a proinflammatory diet may increase the risk of low CRF. The association persisted even after adjusting for various covariates, including race, sex, age, BMI, DM, hypertension, CKD, smoking status, alcohol consumption status, physical activity and Hb.

The CRF reflects the capacity of the heart and lungs to transport oxygen to the mitochondria within skeletal muscles, facilitating energy generation during exercise (2, 22). CRF is increasingly recognized as vital for overall health, significantly contributing to the prevention and management of various chronic diseases (23). CRF serves not only as an indicator of physical fitness but also as a predictor of health outcomes. Multiple studies have emphasized the strong links between low CRF levels and increased risks of CVD, cause-specific mortality, all-cause mortality, and several types of cancer (1, 5). Furthermore, CRF is pivotal in shaping the health of young individuals, influencing a spectrum from their physical condition to their psychological health (24). Research has demonstrated that higher CRF levels in youth are linked to a lower risk of early CVD, better cardiometabolic health, improved mental health, and greater academic achievement (2). In middle-aged, employed men free of CVD, CRF was significantly associated with longevity over four decades, with benefits extending well into later life (25). A large study of US Veterans without known CVD revealed that every 1-MET increase in CRF corresponded to a 16% reduction in the risk of major adverse cardiac events (MACE) (26). This underscores the inverse relationship between CRF levels and cardiovascular risk, highlighting that improving CRF can significantly reduce the likelihood of major cardiac events. A substantial body of evidence supporting the role of CRF in health and disease prevention is compelling (27, 28). These findings demonstrate that CRF is not only an indicator of physical fitness but also a crucial predictor and modifiable risk factor for a wide array of health outcomes.

Inflammation plays a pivotal role in various systemic health issues that can significantly impact CRF. A study revealed that lower CRF was associated with increased CRP levels in women with DM (29). Research addressing metabolic syndrome and its association with inflammation has revealed that the degree of CRF in individuals with metabolic syndrome is inversely related to CRP, IL-6, and interleukin-18 levels (6). Chronic inflammatory responses contribute to the development of atherosclerosis, a key factor in CVD, which thickens and narrows the arterial walls, thereby reducing the heart’s blood supply (30). This same inflammatory process extends to lung health, which impairs lung function and the efficiency of oxygen exchange (31–33). Additionally, inflammation negatively affects muscle cell metabolism and function, decreasing muscle efficiency and endurance. Cytokines such as TNF-α and IL-6, which are associated with inflammation, inhibit muscle growth and repair, leading to muscle strength reduction and exacerbating conditions such as sarcopenia in elderly individuals, where muscle loss and weakness are pronounced (34, 35). These interconnected effects highlight the broad impact of inflammation on vital body systems that govern overall physical performance and health.

Diet is a potent tool for improving systemic inflammation (36, 37). Researchers have developed the DII to evaluate the inflammatory potential of diets on the basis of the levels of anti-inflammatory and proinflammatory foods (19). Studies have demonstrated that the DII is significantly associated with various systemic inflammation markers, including IL-1, IL 2, interferon-gamma, vascular cell adhesion molecule, TNF-α, and procalcitonin (38, 39). Our study focuses on the relationship between the DII and CRF, offering significant insights to the existing literature, which has focused primarily on the associations between the DII and outcomes such as all-cause mortality and CVD. Although current studies (40, 41) have explored how pro-inflammatory diets, indicated by higher DII scores, impact long-term health outcomes and mortality risks, such studies often overlook the direct effects of dietary inflammation on physical fitness. By highlighting the direct association between DII and CRF, our research provides mechanistic insights into how diet-induced inflammation affects physical fitness, enabling the identification of specific dietary components that increase or decrease CRF. This is particularly valuable for developing targeted dietary recommendations to improve cardiovascular and respiratory health.

In this study, we evaluated the link between the DII and the risk of low CRF. Our findings indicate that a rise in the DII is closely linked to an increased incidence of low CRF. Prior research examining the link between the Empirical Dietary Inflammatory Index (EDII) score and low CRF (42) revealed a significant inverse relationship between the EDII score and CRF level. Our study included a larger and possibly more diverse population. This expansion not only enhances the statistical power and robustness of the findings but also increases their applicability to a wider range of demographic groups. In contrast to the referenced study, which utilized the EDII, our investigation employs the DII, noted for its thorough and nuanced method of assessing dietary inflammatory potential. The DII, derived from a comprehensive collection of global dietary data, provides a thorough assessment of various nutrients, such as vitamins, minerals, and bioactive compounds. The comprehensive scope of nutrients is a significant advantage, enabling the DII to provide precise evaluations of the impact of individual dietary components on inflammation (43). Consequently, the DII is especially valuable in epidemiological research, as it allows for precise connections between specific nutrients and the risk of various diseases, including CVD and cancer (44). This capability renders the DII a potent instrument for research endeavors focused on unraveling the intricate interplay between diet and chronic inflammation. The exercise treadmill test (ETT) used in our research offers several distinct advantages, particularly compared with the more complex cardiopulmonary exercise test (CPET) (45). Unlike the CPET, which requires sophisticated equipment to analyze gas exchange and involves more intricate protocols, the ETT can be conducted with minimal equipment, reducing both the cost and the complexity of the setup. This makes the ETT a more feasible option for routine screening and widespread clinical use.

Our research revealed a significant difference between men and women, suggesting that women may exhibit greater sensitivity to the influence of dietary inflammation on CRF. The possible reasons for this phenomenon may include the influence of sex hormones such as testosterone (46), which promote the growth of cardiac muscle and cause men’s hearts to be generally larger than those of women, with thicker ventricular walls and greater myocardial mass (47). This difference results in sex differences in cardiac output. Inflammation can affect the efficiency of the heart’s work, including the contractility of the myocardium and the heart’s filling capacity (48). In individuals with a high inflammatory state, CRF is usually lower because inflammation affects the efficiency of oxygen transport and utilization (49). A high DII may indicate that the anti-inflammatory protective effects of estrogen are diminished in women (50), increasing their sensitivity in terms of cardiorespiratory function. In contrast, men may “resist” the effects of diet-induced inflammation to some extent because of their larger baseline myocardial mass.

The association between the DII and participants’ CRF suggests several important findings. First, the association highlights the potential impact of diet on physical fitness and overall cardiovascular health. A diet with high inflammatory potential, as indicated by a higher DII score, may negatively affect CRF, underscoring the importance of dietary choices in maintaining and improving cardiovascular and respiratory efficiency. Second, public health strategies should prioritize anti-inflammatory diets, particularly for women, to enhance cardiorespiratory health and reduce the risk of associated chronic diseases. Finally, the findings suggest that incorporating dietary assessments such as the DII into routine health evaluations could provide valuable insights into an individual’s risk profile for low CRF and associated health outcomes, allowing for more targeted interventions. Overall, the relationship between DII and CRF underscores the integral role of nutrition in fostering CRF and long-term health.

### Limitations

This research has several caveats that warrant mention. First, its retrospective nature imposes certain limitations, allowing us to infer only associations, not causality, between the DII and low CRF. Additionally, while earlier studies utilized 45 food components to compute the DII, our analysis, drawing on NHANES data, was based on a subset of 28 dietary components. Nevertheless, it has been established that this reduced dietary item count does not affect the DII’s prognostic accuracy. Another point to consider is the self-reported aspects of numerous study variables, which introduces the possibility of recall bias. Finally, the study’s findings may not be universally applicable, given that the dataset predominantly represents the U.S. population.

## Conclusion

A higher DII score is correlated with an increased risk of low CRF in adults. Moreover, sex can impact CRF, with women being more prone to low CRF under comparable DII conditions.

## Data Availability

The datasets presented in this study can be found in online repositories. The names of the repository/repositories and accession number(s) can be found below: https://wwwn.cdc.gov/nchs/nhanes/.

## Footnotes

1
https://www.cdc.gov/

2
https://wwwn.cdc.gov/nchs/data/nhanes/2019-2020/manuals/2020-Physician-Examination-Manual-508.pdf

3
https://wwwn.cdc.gov/nchs/data/nhanes/2009-2010/manuals/lab.pdf
